# Exploring surface cleaning strategies in hospital to prevent contact transmission of methicillin-resistant *Staphylococcus aureus*

**DOI:** 10.1186/s12879-016-2120-z

**Published:** 2017-01-18

**Authors:** Hao Lei, Rachael M. Jones, Yuguo Li

**Affiliations:** 1 Department of Mechanical Engineering, The University of Hong Kong, Pokfulam, Hong Kong, SAR China; 2 Division of Environmental and Occupational Health Sciences, School of Public Health, University of Illinois at Chicago, Chicago, IL USA

**Keywords:** Methicillin-resistant *Staphylococcus aureus*, Hospital, Surface cleaning, Mathematical model, High-touch surfaces

## Abstract

**Background:**

Cleaning of environmental surfaces in hospitals is important for the control of methicillin-resistant *Staphylococcus aureus* (MRSA) and other hospital-acquired infections transmitted by the contact route. Guidance regarding the best approaches for cleaning, however, is limited.

**Methods:**

In this study, a mathematical model based on ordinary differential equations was constructed to study MRSA concentration dynamics on high-touch and low-touch surfaces, and on the hands and noses of two patients (in two hospitals rooms) and a health care worker in a hypothetical hospital environment. Two cleaning interventions – whole room cleaning and wipe cleaning of touched surfaces – were considered. The performance of the cleaning interventions was indicated by a reduction in MRSA on the nose of a susceptible patient, relative to no intervention.

**Results:**

Whole room cleaning just before first patient care activities of the day was more effective than whole room cleaning at other times, but even with 100% efficiency, whole room cleaning only reduced the number of MRSA transmitted to the susceptible patient by 54%. Frequent wipe cleaning of touched surfaces was shown to be more effective that whole room cleaning because surfaces are rapidly re-contaminated with MRSA after cleaning. Wipe cleaning high-touch surfaces was more effective than wipe cleaning low-touch surfaces for the same frequency of cleaning. For low wipe cleaning frequency (≤3 times per hour), high-touch surfaces should be targeted, but for high wipe cleaning frequency (>3 times per hour), cleaning should target high- and low-touch surfaces in proportion to the surface touch frequency. This study reproduces the observations from a field study of room cleaning, which provides support for the validity of our findings.

**Conclusions:**

Daily whole room cleaning, even with 100% cleaning efficiency, provides limited reduction in the number of MRSA transmitted to susceptible patients via the contact route; and should be supplemented with frequent targeted cleaning of high-touch surfaces, such as by a wipe or cloth containing disinfectant.

**Electronic supplementary material:**

The online version of this article (doi:10.1186/s12879-016-2120-z) contains supplementary material, which is available to authorized users.

## Background

Methicillin-resistant *Staphylococcus aureus* (MRSA) has become an important cause of hospital-acquired infections (HAIs) worldwide [1]. MRSA can be transmitted by the contact route [2], which is consistent with the influence of MRSA-contaminated environmental surfaces, equipment, and hands of health care workers (HCWs) on MRSA HAIs [3]. Cleaning and disinfection of environmental surfaces is an obvious intervention that has been found to be important for the control of MRSA in the environment [4, 5]. Optimal cleaning strategies, however, remain uncertain.

Cleaning policies must consider how to clean, where to clean, and when to clean. Cleaning may involve the whole room, or targeted to specific surfaces. While both strategies are recommended by the Health Care Infection Control Practices Advisory Committee (HICPAC), the HICPAC guidelines do not recommend specific cleaning frequency [6]. More cleaning, when done so as to prevent cross-contamination, will always remove more MRSA and other pathogens from environmental surfaces, but additional cleaning incurs costs, including: personnel time [5], consumption of cleaning products, and disruption of patients. In addition, different surfaces play different roles in MRSA transmission in hospitals. Frequent cleaning of surfaces with frequent hand contact (high-touch surfaces) may have a greater benefit than frequent whole room cleaning, and cleaning high-touch surfaces has been shown to reduce contamination on HCWs’ hands [7]. To our knowledge, little research has explored when, and how frequently surface cleaning should be performed in a health care facility.

The objective of this study was to explore cleaning strategies for the control of MRSA transmission to a susceptible patient in an intensive care unit (ICU) at an acute care hospital. MRSA HAIs are a significant burden in this setting and there is a high potential for contact transmission due to severity of illness [8, 9]. In addition, each HCW provides high-intensity care to a small number of patients [10]. Two surface cleaning interventions were studied: whole room surface cleaning and disinfection and wipe cleaning of touched surfaces [11]. Whole room cleaning is typically performed by environmental service workers, and while the frequency is not standardized, this activity was assumed to occur daily. In contrast, wipe cleaning of touched surfaces may be performed by HCWs during care delivery, such that the frequency of this activity is higher than whole room cleaning. Targeted wipe cleaning is a relatively new idea, but wipes have found to reduce bacterial loads on environmental surfaces [12, 13] and to be easy to use [14]. We considered both types of cleaning activities to involve the use of approved disinfectants that are effective against MRSA. We specifically explored: the optimal timing of whole room cleaning, the impact of whole room cleaning efficiency, the optimal frequency of wipe cleaning and allocation to high-touch and low-touch surfaces.

In this study, a mathematical model was used to attain our objectives. Modeling has been successfully applied to evaluate the transmission of HAIs [15–18]. However most of these studies had homogeneous mixing of surfaces, which cannot reproduce the intricacies of the surfaces in a hospital ward. The model used in this study considered two types of surfaces distinguished by the frequency of patient and HCW contacts.

## Methods

### Model overview

**Fig. 1:**
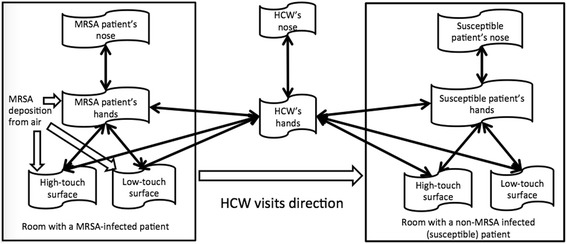
Diagram of MRSA transmission between two hypothetical hospital rooms

Ordinary differential equations (ODEs) were used to describe the change in MRSA concentration on environmental surfaces in two hypothetical patient rooms, two patients and one HCW. Figure 1 displays the ten model compartments, and the equations are described in the Additional file 1: S1. In the room of each patient, two types of surfaces were considered: high-touch surfaces are those subject to frequent contact by patients and HCW, and are typically considered to include bed rails, over-bed tables, call and control buttons, and other surfaces near the patient; and low-touch surfaces are subject infrequent contact by patients and HCW, including curtain, chair and so on [19]. MRSA was considered emitted by one MRSA-infected patient into air as part of epithelial cells [20], onto surfaces in that patients’ room due to deposition from the air and contact by the patient’s contaminated hands. MRSA in the infected patient’s room was transferred to the hands of the HCW upon contact with surfaces or the patient; and the HCW transported MRSA to surfaces to the room of a susceptible patient (not colonized or infected with MRSA) and onto the susceptible patient by contact: this represents transmission by indirect contact. The susceptible patient can also be exposed to MRSA by direct contact with the HCW. The governing ODEs were solved with Matlab (Matlab 2015a, MathWorks, Inc., Natick, Massachusetts).

### MRSA emission rate

MRSA-infected and MRSA-colonized patients are known to emit MRSA into the air in association with epithelial cells [21–24]. The mean emission rate of MRSA into the air from a MRSA-infected patient was estimated to be 325 CFU/min (see Supplemental Material Part 2). That is, the emission rate to air was e_A_ = 325 CFU/min = 19,500 CFU/h. The rate of surface contamination due to deposition of MRSA from the air was calculated as follows. We assumed the volume of a patient room V = 48 m^3^ (4 m × 4 m × 3 m height), with a horizontal surface area equal to the floor area, S = 16 m^2^. We assumed that general ventilation provided six air exchanges per hour [25, 26], equal to a volumetric airflow rate, Q = 288 m^3^/h. The steady-state MRSA concentration in air was assumed to be C_A_ = e_A_ ÷ Q = 67.7 CFU/m^3^. Airborne epithelial cells range in size from 4 to 20 μm, with a median diameter of 14 μm [27]. The deposition loss rate was calculated for the median particle diameter using the observed rate in furnished environments [28]: α = k × r^2^ where k = 0.1375/(h-μm^2^); r is the particle radius (μm) and α is the loss rate (/h). For r = 7 μm, α = 6.74/h. Particles were assumed to be uniformly distributed in the environment, such that the rate of MRSA deposition onto surfaces is *e*_*s*_ = C_A_ × V × α/S = 0.14 CFU/(cm^2^ ‐ h). In addition, the MRSA emission rate on the MRSA-infected patient’s hands *e*_*h*_ was assumed to be equal to *e*_*s*_.

During the visit to the MRSA-infected patient’s room, the HCW would touch the patient’s skin (not including the patient’s hands), which may transfer MRSA bacteria from the patient’s skin to the HCW’s hands. A study found that 0.1 to 3 CFU were transferred per minute from an infected skin site to an HCW’s hands [29]. Given a mid-point emission rate to the HCW’s hands of 1.5 CFU/min (or 90 CFU/h) and uniform distribution on the palm of one hand (150 cm^2^), the mean emission rate from the infected patient to the hands of the HCW was *e*_*hcw*_ = 0.6 CFU/(cm^2^-h).

### Time activity patterns

Contact events transfer bacteria between the compartments: in each contact event, MRSA was considered transferred in both directions (i.e., to and from the two contacting surfaces). The efficiency of the transfer process depends upon the surface types and was not considered reciprocal. We assumed that the HCWs and the patients touched their noses with their fingertips between 09:00 and 21:00. After 21:00, the patient was considered to be asleep and have no hand-to-nose or hand-to-surface contact. The MRSA concentration in the MRSA-infected patient’s nose was assumed to be a constant 250 CFU/cm^2^ [20], consistent with prolonged nasal colonization. For the susceptible patient and the HCW, touching the nose resulted in an increase in the MRSA concentration in their nose, but this was not considered to affect infection status during the simulation period.

The HCW touched environmental surfaces during patient visits between 09:00 and 17:00, with different contact rates on high-touch (χ_hcw-hs_) and low-touch (χ_hcw-ls_) surfaces. Patients also touched the environmental surfaces between 09:00 and 21:00, with different contact rates on high-touch (χ_hs_) and low-touch (χ_ls_) surfaces. After 21:00, the patients slept until 09:00 the next day, and it was assumed that there was no hand-to-surface or hand-to-nose contact during sleep.

We assumed that an HCW worked an 8-h shift starting at 09:00, which on the first day was also set as the time the simulation began. In each hour, the HCW visits the room of the MRSA-infected patient for the first 20 min, visits the room of the susceptible patient for the next 20 min, and then completes the hour at the nurses’ station.

The initial MRSA concentration on the MRSA-infected patient’s nose was assumed to be 250 CFU/cm^2^ [20], and in the other nine compartments, it was assumed to be 0.

### Model interventions

We considered whole room surface cleaning to occur daily in rooms with patients with an infectious disease [20]. In the baseline simulation, we considered whole room cleaning to occur at 8:00, and we tested other times of day to determine the influence on the MRSA levels.

Wiping of touched surfaces was assumed to occur during care delivery, consistent with the study by Plipat et al. [20]. The surface wiping frequency was defined separately for high-touch surfaces (*w*_*hs*_, touch/h) and low-touch surfaces (*w*_*ls*_, touch/h). In the baseline simulation, *w*_*hs*_ = *w*_*ls*_ = 1 per hour, but we considered frequencies as high as 18 per hour, and to differ between the two surface types. The high values are consistent with a modeling study by Plipat et al. [20], in which the wiping frequency was the same as the contact frequency on surfaces, though we consider it unlikely this frequency would actually be performed.

The effectiveness of whole room cleaning and wipe cleaning is driven by the efficacy of the disinfectant, and the completeness with which the work task is performed. An efficacious disinfectant would yield a 2-3 log_10_ reduction in MRSA on surfaces [30]. However, cleaning may be incomplete [31]. Therefore, in the baseline simulation, whole room cleaning and wipe cleaning efficiency were both set as 44%, the product of 91% surface cleaning efficacy [30] and the 48% compliance rate of surface cleaning [32].

For hand washing, we assumed that the patients did not wash their hands. Though HCWs should wash hands regularly during patient care, hand hygiene compliance is usually incomplete [33, 34]. In twelve observation studies in ICUs (see detail in Additional file 1: Supplementary S2.D), the average compliance rate with was 41%. Hand hygiene was considered to remove 90% of bacteria [35], which gives an overall hand hygiene efficiency of 37%, *I*_*h*_ = 0.41 × 0.90 = 0.37.

The variable used to measure the impact of cleaning is the concentration of MRSA in the nares of the susceptible patient and the HCW, which better reflects potential health outcomes than environmental levels [11]. Risk of colonization and risk of infection were not calculated because the dose-response function for infection arising from dermal exposure to *S. aureus* assumes occlusion of the inoculation site for a week, which is not reasonable [36, 37]; and the risk of colonization has only been associated with airborne concentrations of *S. aureus* [22]. However, for relatively low exposures to pathogens, dose is directly proportional to risk [38], which means that reductions in exposure are informative about reductions in risk.

All model parameters are summarized in the Additional file 1: Table S1.

## Results

### MRSA concentration dynamics

**Fig. 2:**
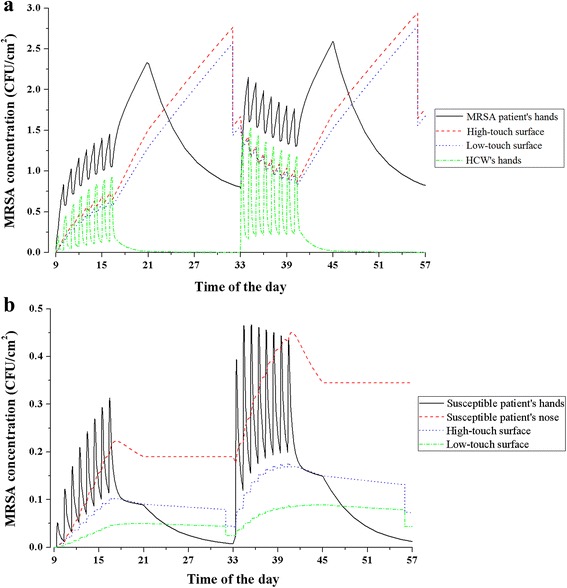
MRSA concentrations dynamics in baseline scenario in 48-h simulation. (**a**) in MRSA-infected patient’s room and HCW’s hands, and (**b**) in susceptible patient’s room. Note difference in scales on y-axis

The MRSA concentration dynamics over the course of two consecutive days, starting at 9:00, are depicted in Fig. 2: (a) reflects the surfaces in the MRSA-infected patient’s room,MRSA-infected patient’s and the HCW’s hands, and (b) reflects the surfaces in the susceptible patient’s room and susceptible patient’s hands and nose. The dynamic pattern is similar on subsequent days (data not shown). Briefly, in both rooms, variation from 9:00 to 17:00 is driven by visits by the HCW, after which MRSA emission into the air and onto surfaces continues while the patient sleeps. Whole room cleaning at 9:00 on day 2 results in a dramatic reduction on high-touch and low-touch surfaces.

### Wipe cleaning

**Fig. 3:**
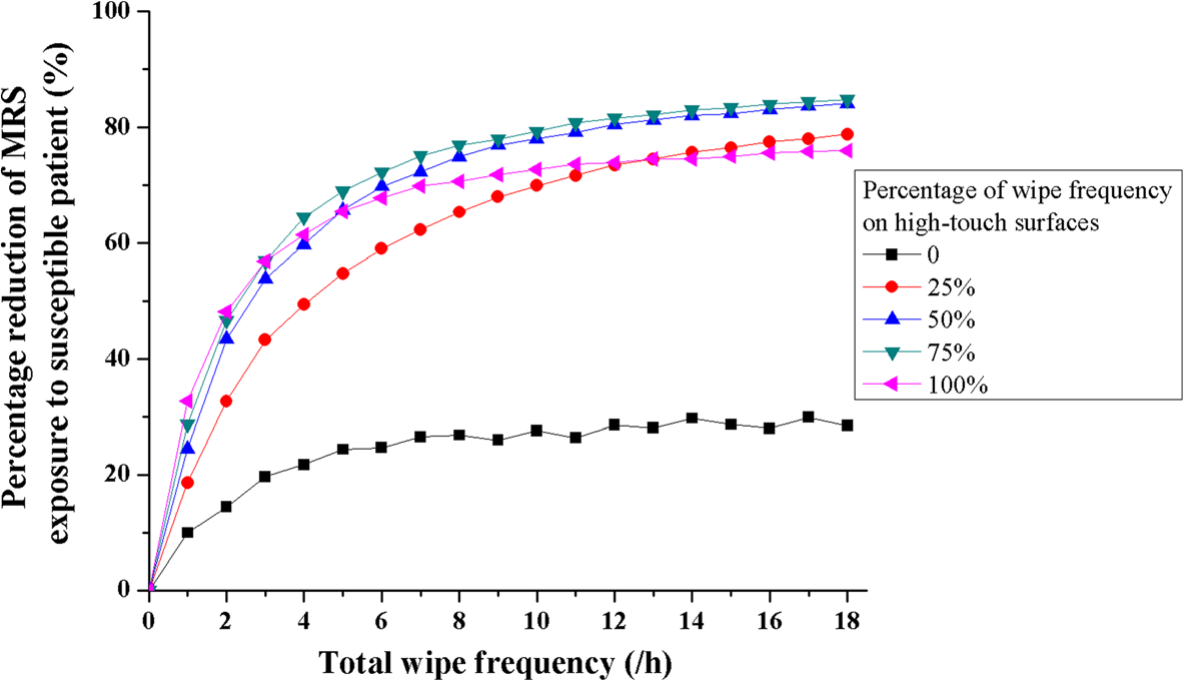
MRSA reduction on susceptible patient’s nose due to surfacing wiping frequency increase, with various percentages on high-touch surfaces

Wipe cleaning of both high- and low-touch surfaces provides substantial reduction in the exposure of the susceptible patient to MRSA (measured as MRSA in the susceptible patient’s nose), relative to no surface wiping (Fig. 3). The gains achieved by increasing the frequency of surface wiping begin to diminish above three per hour. A total wiping frequency of three per hour results in, at most, a 57% reduction in the MRSA exposure. And increasing the wipe frequency to six per hour results in, at most, a 72% reduction in the MRSA exposure, or a 15% increase for double the work (in Fig. 3). There is a clear advantage to wiping high-touch surfaces alone, or in combination with low-touch surfaces, relative to simply wiping low-touch surfaces, but at low wipe frequencies (1-3 per hour), there is an advantage to wiping only high-touch surfaces.

**Fig. 4:**
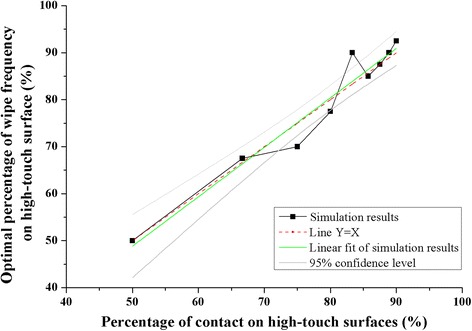
Optimal wiping frequency on high-touch surfaces with different percentages of hand-to-surface contact on high-touch surfaces

The allocation of surface wipes between high- and low-touch surfaces that minimizes the exposure of the susceptible patient is approximately equal to the relative to the total contact frequencies to these surface types by patients and HCW combined (Fig. 4). The linear relationship fitted to the simulation results has slope 1.05 (standard error = 0.09; R^2^ = 0.94), though the 1:1 line falls within the 95% confidence interval of the fitted line. We think that small this difference is due to the computation error of the numerical method. In addition, realistically, since the total contact frequencies (patients and HCW combined) are much less than 100, it is not possible to implement small changes in allocations, so it is reasonable to recommend wiping high- and low-touch surfaces in proportion to the frequency with which they are touched, when the total wipe frequency is high (more than 3 times per hour).

We found that the optimal allocation of wipe frequency between high- and low-touch surfaces was not sensitive to changes in: the MRSA pathogen transfer efficiency between different compartments during contact, the MRSA inactivation rate, the MRSA emission rate to environmental surfaces and hands, patient and HCW contact rates on environmental surfaces, hand hygiene effectiveness, or to surface cleaning effectiveness (see Additional file 1: Table S3).

### Whole room cleaning

**Fig. 5:**
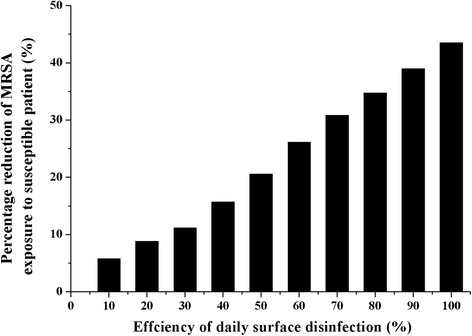
MRSA reduction on susceptible patient’s nose with various disinfection efficiencies

In the baseline scenario, the efficiency of whole room cleaning and disinfection was assumed to be 44%. Figure 5 shows the effects of cleaning and disinfection efficiency in reducing the MRSA concentration in the susceptible patient’s nose, relative to no surface wiping and no surface disinfection. As expected, increasing efficiency reduces exposure linearly, but 100% efficiency reduces exposure by only 44%.

**Fig. 6:**
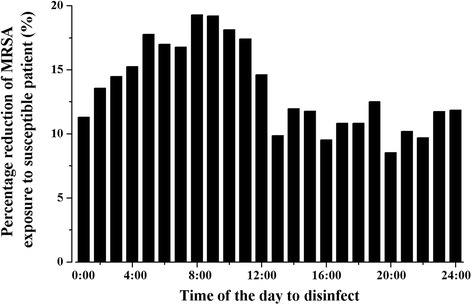
MRSA reduction on susceptible patient’s nose with disinfection performed at various times

The greatest reduction (19%) in the MRSA concentration on the nose of the susceptible patient, relative to no whole room cleaning or wipe cleaning, occurs when cleaning is performed when the HCW begins to visit patients for the day, at 09:00 (Fig. 6). If whole room cleaning is performed around 09:00 (between 07:00 and 11:00), the reduction in the MRSA concentration is near its greatest point (from 17 to 19%). If disinfection is performed at any time during the evening after the HCW ceases activities for the day (between 17:00 and 24:00), the average reduction in MRSA exposure for the susceptible patient is only 11%.

## Discussion

In this study, we used a mathematical model to explore environmental surface cleaning practices to prevent the contact transmission of MRSA between two patients who are cared for by the same HCW in a hospital ICU. Though we have simplified the human activity patterns and environment of an ICU, the model captures critical behaviors and surfaces relevant to the contact transmission of infectious diseases. For example, we consider a 1:2 HCW to patient ratio, which is consistent with staffing in the ICUs [39]. This model is not fully generalizable to all hospital settings, however, due to the use of single-patient rooms and the high-intensity of patient care. However, we feel the general findings about the value of wipe cleaning and timing of whole room cleaning are likely to hold true in other settings.

### Verification of the model

There is always concern that the model has insufficient fidelity to the real world. To increase confidence in our model, we simulated the prospective crossover study of Dancer et al. [5]. In this study, enhanced cleaning was performed between 7:30 and 15:30, and included 1-3 additional daily cleanings of clinical equipment, high-touch sites near the patient and at the nurses’ station, and was found to produce a 32.5% reduction of microbial contamination on the touched sites.

In testing our model, baseline cleaning was represented by whole room cleaning performed once per day (at 08:00) and two enhanced cleanings were performed at 10:30 and 13:30 (uniformly distributed between 07:30 and 15:30). The enhanced cleaning was modeled as cleaning high-touch surfaces, or as whole room cleaning. The compliance rate with the enhanced cleaning was assumed to be 80%, higher than the compliance rate of the hospital daily cleaning (48%), since these cleanings were performed by designated personnel. Enhanced cleaning of high-touch surfaces reduced mean MRSA concentrations on high-touch surfaces in both patient rooms by 31.2% relative to baseline. Enhanced whole room cleaning reduced mean MRSA concentrations on high-touch surfaces in both patient rooms by 35.3% relative to baseline, and that the mean reduction on both high- and low-touch surfaces was 36.6% relative to baseline cleaning. These values agree well with the 32.5% reduction from field study.

### Where to clean?

We found that high-touch surfaces should be cleaned more frequently than low-touch surfaces to reduce MRSA transmission via the contact route (Fig. 3). There remains value, however, to cleaning low-touch surfaces when the total wipe frequency is more than 3 times per hour. This finding is consistent with evidence showing the importance of high-touch surfaces in the transmission of pathogens in hospitals [19, 40], and recommendations by others to clean near-patient sites in hospitals [5, 41, 42].

We found that when the frequency of wipe cleaning is relatively low (i.e., less than four times per hour), the cleaning workload should be allocated to high-touch surfaces only. When the wipe cleaning frequency is relatively high (more than three time per hour), the wipe cleaning should be allocated to high- and low-touch surfaces in proportion to the frequency with which they are touched, which coincides with recent recommendations from the HICPAC regarding the cleaning and disinfection of high-touch surfaces (e.g., doorknobs, bed rails, light switches, and surfaces in and around toilets in patients’ rooms) on a more frequent basis than minimally touched surfaces [6].

### How to clean?

We found that frequent surface wiping, even with relative low efficiency (44%) and frequency (three times per hour), is more effective than whole room cleaning. Even with 100% disinfection efficiency, whole room cleaning performed once per day would only reduce the MRSA on the susceptible patient’s nose by 44%, which is consistent with findings another modeling study [20]. However, wiping high-touch surfaces only three times per hour with 44% efficiency by HCWs during the 8-h shift can lead to a 57% reduction of the MRSA on the susceptible patient’s nose. One reason for the relatively poor impact of whole room cleaning is that surfaces are rapidly recontaminated after disinfection [43, 44], which suggests the value a disinfectant with residual anti-microbial activity.

Cross-contamination poses a challenge to whole room and wipe cleaning, and can occur readily if the same cleaning cloth or tool is used on multiple surfaces [30, 45]. Cross-contamination of MRSA during cleaning was not considered in this study, and represents a limitation of the model that would tend to underestimate the MRSA concentration on surfaces of all types. In practice, cross-contamination is managed by the frequent disposal of cleaning cloths [6].

### When to clean?

We found that wipe cleaning should be performed during care activities, and that whole room cleaning is optimally performed in the morning, prior to the first health care visit of the patient by the HCW. The reduction of the MRSA on the susceptible patient’s nose when disinfection is performed just before the health care visit is 1.7 times that when disinfection is performed after the health care visit and before midnight (19% vs 11%).

In this study we have not specifically considered the potential for MRSA colonization or infection in the susceptible patient or HCW, but have considered MRSA exposure of the susceptible patient. Exposure analysis is an essential step in the quantitative risk assessment framework, and moves us towards improved understanding of infection transmission [20]. Low exposures are typically proportional to infection risk [38], so reductions in exposures would yield a proportional reduction risk, even when the probability of infection is not quantified. We considered estimating the risk of infection from the MRSA exposure data, but there are significant knowledge gaps about the dose-response function and the relevant time window of exposure that would add to the uncertainty in the results.

## Conclusions

In this study, we constructed a mathematical model to study the MRSA concentration dynamics in a hypothetical hospital environment, and the performance of two cleaning interventions – whole room cleaning and wipe cleaning of touched surfaces. Daily whole room cleaning was found to be less effective at controlling MRSA transmission via the contact route in hospital, even with 100% cleaning efficiency, than frequent wipe cleaning of touched surfaces during healthcare delivery. When wipe cleaning is frequent, cleaning should be allocated to surfaces in proportion to the frequencies with which they are touched.

## Additional file

Additional file 1: Supplementary material. (DOCX 304 kb).

## Abbreviations

HAIs: Hospital-acquired infections
HCW: Health care worker
HICPIC: Health care infection control practices advisory committee
MRSA: Methicillin-resistant *Staphylococcus aureus*
ODEs: Ordinary differential equations
